# Altered Gene Expression Profiles of Wheat Genotypes against Fusarium Head Blight

**DOI:** 10.3390/toxins7020604

**Published:** 2015-02-16

**Authors:** Ayumi Kosaka, Alagu Manickavelu, Daniela Kajihara, Hiroyuki Nakagawa, Tomohiro Ban

**Affiliations:** 1 Kihara Institute for Biological Research, Yokohama City University, Maioka 641-12, Totsuka, Yokohama 244-0813, Japan; E-Mails: ayumikosaka25@hotmail.com (A.K.); manicks@yokohama-cu.ac.jp (A.M.); ka_danik@yahoo.com.br (D.K.); 2 Vascular Biology Laboratory, Heart Institute (InCor), University of Sao Paulo, School of Medicine, Av. Eneas C Aguiar, 44-Annex 2, 9th floor, Sao Paulo 05403-900, Brazil; 3 National Food Research Institute, National Agriculture and Food Research Organization, 2-1-12 Kannondai, Tsukuba 305-8642, Japan; E-Mail: hironkgw@affrc.go.jp

**Keywords:** wheat, *Fusarium graminearum*, microarray, molecular response, detoxification, local and systemic response

## Abstract

*Fusarium graminearum* is responsible for Fusarium head blight (FHB), which is a destructive disease of wheat that makes its quality unsuitable for end use. To understand the temporal molecular response against this pathogen, microarray gene expression analysis was carried out at two time points on three wheat genotypes, the spikes of which were infected by *Fusarium graminearum*. The greatest number of genes was upregulated in Nobeokabouzu-komugi followed by Sumai 3, whereas the minimum expression in Gamenya was at three days after inoculation (dai). In Nobeokabouzu-komugi, high expression of detoxification genes, such as multidrug-resistant protein, multidrug resistance-associated protein, UDP-glycosyltransferase and ABC transporters, in addition to systemic defense-related genes, were identified at the early stage of infection. This early response of the highly-resistant genotype implies a different resistance response from the other resistant genotype, Sumai 3, primarily containing local defense-related genes, such as cell wall defense genes. In Gamenya, the expression of all three functional groups was minimal. The differences in these molecular responses with respect to the time points confirmed the variation in the genotypes. For the first time, we report the nature of gene expression in the FHB-highly resistant cv. Nobeokabouzu-komugi during the disease establishment stage and the possible underlying molecular response.

## 1. Introduction

Fusarium head blight (FHB) is a destructive disease in wheat that is caused by *Fusarium graminearum*, which infects during the flowering stage and is favored by warm and highly humid climates. One of the main problems from the infection is the accumulation of mycotoxins, such as deoxynivalenol (DON), in the grains during the spread and development of the fungus. DON results in feed refusal or poor weight gain in animals, as well as immunological problems in humans [1]. DON-3-glucoside (D3G), a plant metabolite of DON, has also been detected in cereal grains and cereal-based products [2]. The development and utilization of wheat cultivars with FHB resistance is one of the most important research and breeding objectives in the world, and it has been recognized as one of the most economical, environmentally safe and effective strategies for the control of the disease [3]. To date, two basic, resistant phenotypes have been well studied: type I, which is resistant to primary infection, and type II, which is resistant to disease spread [4]. However, it has been reported that there are three additional types of resistance: resistance to DON accumulation [5], resistance to kernel infection and tolerance of yield loss. These last two types are rarely reported, because their underlying mechanisms are unclear. Sumai 3, a Chinese resistant wheat cultivar, is known to carry stable Quantitative Traits Loci (QTLs) for type I and II resistance that have been well-validated and are highly reproducible [6,7]. Japanese cultivar Nobeokabouzu-komugi (hereafter, Nobeokabouzu) is also an important source of high FHB resistance, particularly for low levels of mycotoxin contamination [8]. However, the study and utilization of Nobeokabouzu has not included the examination of its resistance genes. Additionally, the underlying molecular mechanism of this new source of resistance and the molecular differences between this cultivar and other resources are not yet clear, so they should be studied for future utilization.

Gene expression studies are vital for understanding the establishment of disease and the relevant defense response mechanisms. Several plants have known expression of genes, such as UDP-glycosyltransferase (UGTs), which play a major role in the detoxification process by conjugating DON with glucose into D3G [9,10,11,12]. Others, such as ABC transporters, multidrug resistance-associated protein (MRP), multidrug-resistant protein (MDR) and pleiotropic drug-resistant protein (PDR), are also known to increase expression in response to *F. graminearum* and to confer resistance by playing the role of a pump to export fungal mycotoxin from the cytoplasm [13,14,15,16,17]. Previous reports indicate that the ABC transporter, UGTs and protease inhibitor genes associated with the defense mechanism against fungal virulence factors are apparently active in different resistant genetic backgrounds [17].

Here, we used a comprehensive transcriptomic approach to investigate genes that respond to *F. graminearum* in highly resistant (Nobeokabouzu-komugi), resistant (Sumai 3) and susceptible (Gamenya) wheat cultivars. Through systematic data analysis, the expressed genes can be classified into three major categories, commonly expressed genes, host genotype-specific genes and unique FHB resistant genes, and then further classified into 11 gene classes. By comparing each class of the induced genes in the three response categories, it is possible to examine wheat *Fusarium graminearum* (*F.g*) response during disease establishment. The genes involved in defense response mechanisms can then be categorized into three new functional groups: systemic response, local response and detoxification. We also explain the reason for the highly resistance nature of Nobeokabouzu against FHB.

## 2. Results

### 2.1. Phenotyping

**Figure 1:**
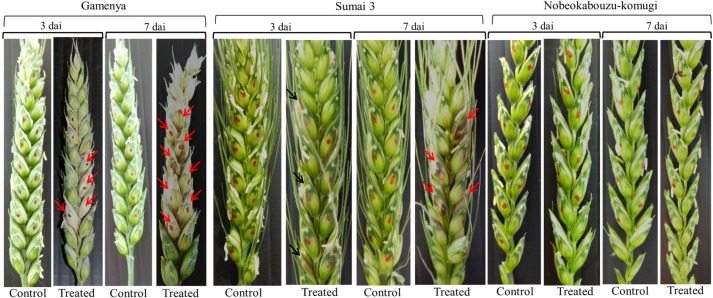
Fusarium head blight (FHB) symptoms among the genotypes. Photographs of water-inoculated control (Control) and *F. graminearum*-inoculated (Treated) wheat plants at three and seven days after inoculation (dai) in susceptible (Gamenya), resistant (Sumai 3) and highly resistant (Nobeokabouzu-komugi) genotypes. Inoculated spikelets are marked with red and black dots. The red arrows indicate necrotic spikelets in Gamenya and brownish necrosis in Sumai 3. The black arrows indicate spikelets where brownish necrosis started in Sumai 3.

**Figure 2:**
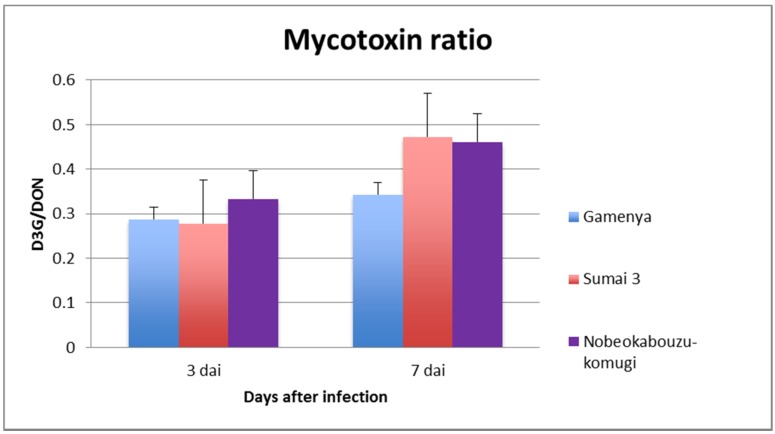
Mycotoxin analysis of wheat genotypes. The ratio was calculated from the absolute value of deoxynivalenol (DON) and DON-3-glucoside (D3G) (µg/kg). DON and D3G were analyzed, and the ratio was interpreted among three genotypes at two different time points.

Figure 1 shows the clear differences in disease FHB progression in the three genotypes; the symptoms appeared quite early in Gamenya, followed by Sumai 3, and no symptoms were observed in Nobeokabouzu. In the case of mycotoxin analysis, by using the DON and D3G concentration among the genotypes and time points (Figure S1), D3G/DON was taken into consideration and the results compared. Comparing three- and seven-day samples, there was not much change in the mycotoxin level of Gamenya (*t*-test, *p* = 0.152), whereas there is significant change of the content occurring in both resistant varieties (*t*-test, *p* < 0.05) (Figure 2). Especially Nobeokabouzu showed a high level of detoxification in 3 dai itself, by means of showing significant conversion of DON into D3G when compared to Sumai 3.

### 2.2. Comparative Gene Expression of Three Wheat Genotypes to F. graminearum

**Figure 3:**
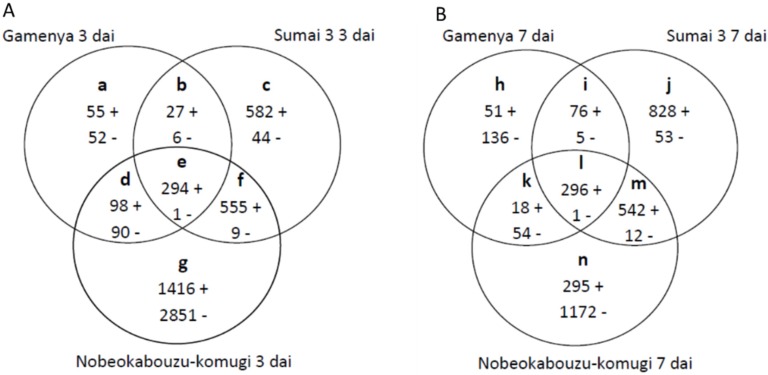
Venn diagram showing the number of wheat *F. graminearum*-responsive genes at three (**A**) and seven (**B**) days after inoculation (dai). Specific and common genes were categorized from the non-section and inter-section, respectively. + and − signs indicate up- and down-regulated genes, respectively.

Comparing the overall gene expression among the genotypes, both the up- and down-regulated genes were abundant in highly resistant Nobeokabouzu (Figure 3A,B, Sections g and n) whereas, in Sumai 3 (Figure 3A,B, Sections c and j), the up-regulated genes were more abundant than the down-regulated genes. In Gamenya, both were minimum expressed (Figure 3A,B, Sections a and h). This result showed the difference in gene expression abundance among the genotypes and laid the path for further studies. Data analysis by Venn diagram enabled the identification of commonly expressed (common *F.g*-responsive) genes (Figure 3, Sections e and l) and genotype-specific *F.g*-responsive genes at 3 dai (Sections a, c and g) and 7 dai (Sections h, j and n). Genes that were commonly expressed in two genotypes against the pathogen were derived (Sections b, f and d and i, m and k for 3 and 7 dai, respectively). Those genes that were expressed in both resistant wheat genotypes are possible FHB-resistant candidate genes (Sections f and m) related to the early or late stages of infection. Comparison of differentially expressed gene abundance at 3 dai showed high expression of the upregulated genes in Nobeokabouzu followed by Sumai 3 and Gamenya (Figure 3A, Sections g, c and a, respectively), whereas at 7 dai, expression was greater in Sumai 3, followed by Nobeokabouzu and Gamenya (Figure 3B, Section j, n and h, respectively). At both treatment time points, the expression level of Gamenya was minimal when compared to the other two genotypes, but a varied gene family was observed between the FHB-resistant wheat genotypes Nobeokabouzu and Sumai 3. This result further helped to show the different resistance and defense responses and/or genes in our selected resistant genotypes.

**Figure 4:**
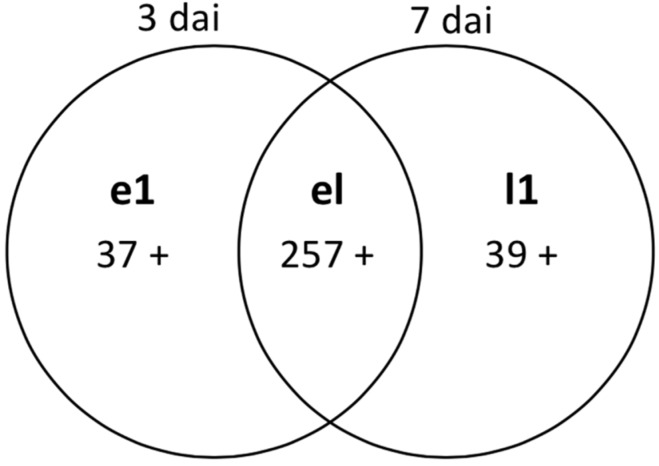
*F. graminearum*-responsive related wheat genes expressed specifically at 3 dai (e1), 7 dai (l1) and both time points (el).

Gene expression analysis was carried out for the common *F.g*-responsive genes (Figure 3, Sections e and l) to determine the gene class, as well as the level of expression among the genotypes (Figure 4 and Table S1). The abundance of genes expressing within 3 dai was 37 (Section e1) and included those related to cell wall defense, such as xylanase inhibitors, the genes involved in detoxification and secondary metabolism, such as glutathione S-transferase (GST), and other miscellaneous defense-related genes, such as the protein related to pathogenesis and the genes related to transcription/signaling. At 7 dai, 39 genes were identified (Section l1), which are related to detoxification, proteolysis, defense, transcription/signaling and uncategorized genes. The number of common transcripts expressing at both treatment time points was 257 (Section el), including the JA- and ET-related genes, such as the genes involved in lipid metabolism and TAG (triacylglycerol) synthesis, AMPs, such as the serine protease inhibitor and thaumatin-like protein, peroxidase (POD) and genes related to cell wall defense, such as endochitinase and glucan-β 1,3-glucosidase. Genes related to detoxification processes, such as the UDP-glycosyltransferase family (UGTs) and the ABC transporter, were also found, along with miscellaneous defense-related genes and genes involved in transcription/signaling and hormone metabolism (Table S2).

### 2.3. Genotype-Specific Differentially Expressed F.g-Responsive Genes

**Figure 5:**
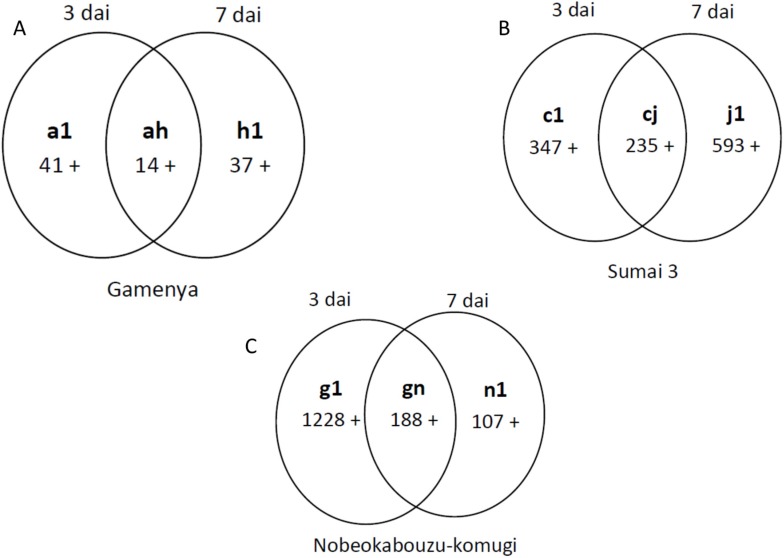
Number of wheat differentially expressed genes in individual genotypes. (**A**) Gamenya at 3 dai (a1), 7 dai (h1) and both time points (ah); (**B**) Sumai 3 at 3 dai (c1), 7 dai (j1) and both time points (cj); (**C**) Nobeokabouzu at 3 dai (g1), 7 dai (n1) and both time points (gn).

Among the time points, no difference in transcriptome abundance was found in Gamenya, whereas, in Sumai 3 and Nobeokabouzu, this was high at 7 and 3 dai, respectively (Figure 5A–C). At 3 dai, *F. graminearum*-responsive up-regulated genes were 41, 347 and 1228 in Gamenya, Sumai 3 and Nobeokabouzu, respectively, while they were 37, 593 and 107 at 7 dai. In the three genotypes, the numbers of genes expressed for both treatment time points were 14, 235 and 188, respectively. Here, clear differences in the numbers of upregulated genes were found between the FHB-resistant and susceptible wheat cultivars, and transcript abundance expression was high in Nobeokabouzu and Sumai 3, but lower in Gamenya. Furthermore, between the two resistant wheat cultivars, Nobeokabouzu expressed more genes at 3 dai (1228 differentially upregulated *F.g*-responsive genes) while Sumai 3 expressed more at 7 dai. The genes involved in detoxification processes, such as UGTs, GST and cytochrome P450, were the most expressed (Table S3), while, compared to Gamenya and Sumai 3, the number and transcript abundance of other *F.g*-responsive genes were also very high.

To clarify the difference in functional response among the genotypes, the expressed genes were classified into three functional groups (I, systemic defense; II, local defense; III, detoxification) (Figure S2, Table S3). In the susceptible Gamenya cultivar, few genes related to local defense, such as nsLTP, thionin (AMP gene class) and gene classes 5–8, were detected. This indicates minimal expression of defense-related genes in this susceptible genotype. In the case of Sumai 3, the expression of local defense-related genes (Group II) was especially high in the later stage of infection, even though all three gene groups were identified ,and the expression level of certain genes, such as thionin (AMP gene class), increased significantly between 13- and 60-fold. In Nobeokabouzu at 3 dai, the expression level of the genes in all of the groups was high, which indicates the quick reaction of this genotype induced by fungal attack. The key defense genes identified were CYP450s, ABC transporters, UGTs, GST, 1-aminocyclopropane-1-carboxylate synthase protein (ACC synthase), several GDSL-like lipases, POD, AMPs, the ubiquitin family, genes related to cell wall defense and PAL. As functional members of the detoxification group (Group III), 17 UGT genes were later found to be positioned in different chromosomes by *in silico* analysis (Table S4). Among these genes, one (Probe ID: 19091; Accession Number GU170357.1) showed significantly high expression compared to the others (68-fold). In addition, many GSTs, volatile phenylpropanoids, cytochrome P450s, ABC transporters and MDR genes were identified. The ACC synthase transcript was significantly highly expressed (46-fold change), and it is the main gene involved in lignin biosynthesis in the systemic defense-related group. Of the genes assigned to Group II, the eukaryotic aspartyl protease family genes showed high expression (57-fold), the role of which in response to *F. graminearum* is unknown, and many of the ubiquitin family genes, particularly Probe ID: 5056 (Accession Number XM006655312), were expressed 98-fold. One of the POD genes (Probe ID: 19329; Accession number X56011.1) was shown to have transcript abundance of 95-fold. However, at 7 dai, in Nobeokabouzu, the number of genes related to Groups II and III was drastically reduced. This was a unique temporal gene expression pattern in response to the pathogen found in the highly resistant genotype.

### 2.4. Confirmation of Gene Expression by Quantitative Real-Time PCR

**Figure 6:**
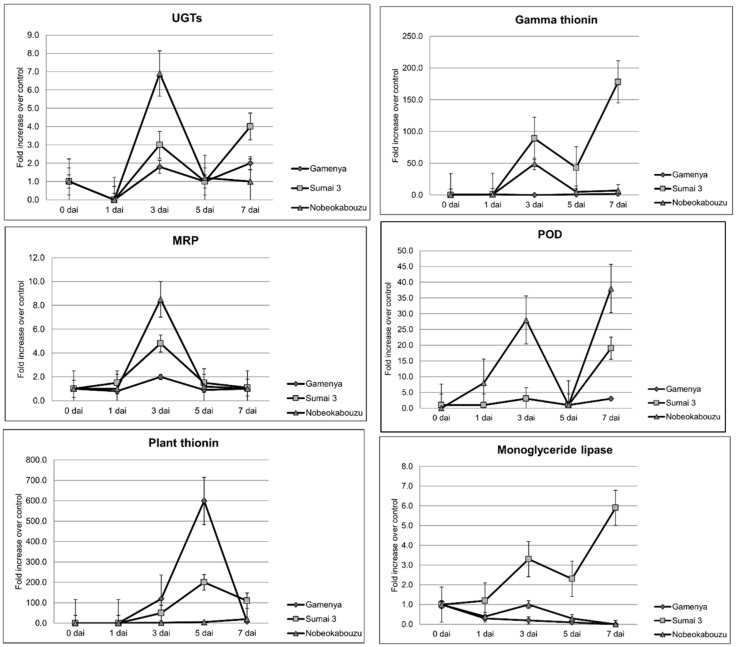
Confirmation of the expression of selected genes in three wheat genotypes by real-time PCR. The expression of each gene is defined as the fold change normalized to the reference gene, GADPH, and relative to the mock-treated control sample. UGTs, UDP-glucosyltransferase; MRP, multidrug-resistant protein; POD, peroxidase; dai, days after inoculation; Nobeoka, Nobeokabouzu-komugi. As shown in Table 1, the gene IDs are: UGT, 15510; MRP, 8976; plant thionin, 23363; gamma thionin, 8436; peroxidase, 19329; monoglyceride lipase, 7118.

In total, six genes were selected, and their differential expression was confirmed (Figure 6). The genes were mainly selected with respect to their specific expression level in either a specific genotype or time point. Although many genes were identified, detoxification gene UGT showed very high expression in Nobeokabouzu and was selected and confirmed (Figure 6A). The role and expression of MRP was reported in a previous study [14]. Here, we have confirmed the extended role of both in Nobeokabouzu (Figure 6B). The remaining four genes were also selected, but were specific to the genotypes. In the case of plant thionin, which is specific to Gamenya at 3 dai, maximum expression was observed at 5 dai (Figure 6C). Gamma thionin expression was similar to the microarray result (Figure 6D). In the case of POD, Nobeokabouzu showed high expression at 3 dai, a decrease at 5 dai and reached maximum expression at 7 dai. In Sumai 3, the expression level was lower and started to increase at 7 dai. However, in Gamenya, the expression level was maintained at a minimum, as expected (Figure 6E). Monoglyceride lipase gene expression was high in Sumai 3 at 7 dai, while it was minimal and showed no concordance with the microarray data in Nobeokabouzu (Figure 6F).

## 3. Discussion

This study was carried out to determine the differential responses of two resistant and one susceptible wheat genotypes against *F. graminearum*. Although many previous transcriptomic studies have attempted to find the difference in the expression of genes related to the FHB-resistant phenotype, all targeted either one or a few major loci by developing Near Isogenic Line (NIL) populations and/or selecting only one resistant and one susceptible cultivar [17,18,19,20,21]. In addition, a previous study using QTL analysis reported that the possibility of FHB resistance is quantitative in nature [22]. Hence, it was necessary to analyze the genome-wide gene expression of the FHB response in wheat genome. Furthermore, we have, for the first time, included a highly resistant variety in our study, Nobeokabouzu, which exhibits low levels of mycotoxin contamination. The DON concentration at 3 and 7 dai was 10.3 ppm and 43.5 ppm, respectively. The concentration is considerably high compared to a previous study [8]. One reason might be the methodology difference, where we have used high precision LC-MS/MS, whereas ELISA was used in the other study. Another specific aim is to compare the different defense responses to the much studied cv. Sumai 3.

Systematic data analysis allowed us to see the difference in the molecular response among genotypes at different time points. Previous *F. graminearum*-related transcriptomic studies have been reported for Sumai 3 and have mainly found the genes with a defense function [17,23,24,25]. In our study, Sumai 3 showed a wide range of genes related to systemic defense (Group I), local defense (Group II) and detoxification (Group III) that were identified as being related to FHB resistance. However, the expression of the local defense genes was higher than other groups (Figure S2). The genes for local defense are gamma thionin, POD and genes related to cell wall defense, such as the pectinase inhibitor, the fungal xylanase inhibitor and the fungal cell wall degradation inhibitor. Compared to 3 dai, transcriptome abundance of differentially expressed genes related to local defense drastically increased at 7 dai, especially gamma thionin (*in silico* chromosome locations 1DS, 4DL and 5BL), nsLTP (4A and 5A), POD (2BS and 3AS) and endochitinase. In a previous report [26], the POD transcript in Sumai 3 was observed as early as 6 h after inoculation and continued to accumulate until 2 dai together with PR-2 (β-1,3-glucanase), PR-3 (chitinase) and PR-5 (thaumatin-like protein). This time interval coincided with conidial germination over the surface of the glume, the beginning of hyphal contact with the stomata and the development of thickened hyphae along the stomatal rows, followed by intracellular hyphae growth in the parenchyma tissue with fungal sporulation [26]. In our study, the expression of POD was high at 7 dai (Figure 6E). During the same time interval, PR-5 (LTP) and AMP expression was also observed. In addition to the genes classes mentioned above, Gottwald *et al.* [17] reported the consistent expression of the serine protease inhibitor (AMPs), which seems to be a candidate resistance gene [17]. Therefore, the mentioned gene classes are possible candidates for FHB resistance in Sumai 3. Another identified transcript, endochitinase, attacks the main structural component of fungal cell walls, the chitin molecules. Chitinase and glucanase in wheat, either directly (via the degradation of the fungal structural barrier) or indirectly (via the activity of fungal cell wall degradation products), play a role in defense and resistance against *F. graminearum* or *F. culmorum*. The identified gene classes and their role in molecular response (local defense) in a timely fashion (7 dai) illustrated the response of Sumai 3 against *F. graminearum*.

The most interesting case was Nobeokabouzu, a highly resistant genotype, in which a broad expression of genes related to the detoxification process, followed by systemic (Group I) and local defense (Group II), was observed at an early stage of infection (3 dai). However, the expression was drastically reduced at 7 dai. The remarkably high expression of transcription involved in the detoxification process, such as the UGTs, CYP450s, ABC transporters and MRP observed in Nobeokabouzu, explains the low level of mycotoxin contamination in Nobeokabouzu, because of the function of those detoxification genes (Group III) [16,27]. This was well correlated with the DON and its conjugate D3G analysis. In addition, in this study, we found a high detoxification process by means of increasing D3G in Nobeokabouzu compared to other genotypes. The D3G/DON ratio among two resistant genotypes, however, did not show a clear significant difference between their UGT gene expression level and D3G contents, either due to post-transcriptional gene modification or the need for more time point samples to confirm. UGTs are encoded by a large gene family with approximately 100–150 members in different plant species [26]. The first UGT capable of detoxifying DON (DOGT1, AtUGT73C5) was identified in *A. thaliana* [13]. In wheat, two UGT genes thought to be involved in DON detoxification, Ta.23272.1.S1_at and TaUGT3 [17], were originally cloned from the FHB-resistant cv. Wangshuibai [12]. In our study, we identified many UGT genes with different expression levels, indicating that many UGT genes are expressed in response to *F. graminearum*. *In silico* mapping analysis showed that UGTs were aligned on already known FHB resistance QTLs. This result indicates the possibility of finding new FHB resistance candidate UGTs or the importance of many UGTs to confer FHB resistance in Nobeokabouzu. One gene among the identified UGTs showed significantly high expression, which was confirmed with real-time PCR. This result further supported the possibility of an early, time-bound detoxification process. However, the substrate specificity of individual genes must be studied to understand their real function with respect to detoxification. Other genes with detoxification features were ABC transporters, such as pleiotropic drug resistant 5 (PDR5-like), which is a well-known, DON-resistant candidate gene. It is like a plasma membrane ABC transporter that co-segregates with the DON-resistant QTL Qfhs.ndus-6BS that is located on 6BS [16]. In our study, the detoxification role of the PDR5-like ABC transporter was shown in both resistant genotypes. Additionally, Nobeokabouzu-specific ABC transporters were identified in this study, and their expression levels were high at 3 dai. Their identification and distribution among chromosomes (*in silico*) indicated the possibility of finding new ABC transporter, PDR5-like genes that differ from known PDR5-like genes. Nobeokabouzu is known to have a close genetic relationship with FHB-resistant Asian landrace sources, such as Nyubai (Japanese landrace) and Wangshuibai (Chinese landrace) [27]. It is also known that FHB resistance in Nyubai is mainly accomplished through the maintenance of cell wall thickness through the deposition of metabolites derived from the phenylpropanoid pathway and not by the conversion of DON to the less toxic D3G [28]. Our results demonstrated the possible defense response mechanism of Nobeokabouzu, which is mainly through DON detoxification in addition to increasing cell wall thickness. This response mechanism is relatively new when compared to either Sumai 3 or the other highly resistant Asian sources. In addition, interestingly, in Nobeokabouzu, the downregulated genes was abundant when compared to other cultivars. However, there were no known resistant- or susceptible-related genes (data not shown), and basically, histone-related genes and hypothetical proteins were found. Therefore, further study will be required in order to clarify the role of those genes as *F.g* responsive.

In the susceptible Gamenya genotype, the gene expression response to *F. graminearum* inoculation was mostly uniform among the time points, except for a few genes, such as POD, glucan endo-1 and 3-β glucosidase (cell wall defense gene class), for which the expression increased at 7 dai. The similar mycotoxin content (DON and D3G) among time points supported the gene expression data. In a nutshell, the limited gene classes and the delayed expression of genes related to defense allowed for initial fungal penetration, and this makes Gamenya susceptible.

## 4. Materials and Methods

### 4.1. Plant Materials

The study was conducted using three bread wheat genotypes. Nobeokabouzu-komugi (Nobeokabouzu) is a Japanese landrace cultivar showing high resistance to FHB and mycotoxin contamination [8]. Sumai 3 is a Chinese variety that possesses type I and II resistance [29,30], and Gamenya is a highly susceptible variety [14]. Homozygous seeds were taken from our laboratory for the study.

### 4.2. F. graminearum Inoculation and Sampling

Plants were grown in a greenhouse under uniform conditions. During early anthesis of each spike, single florets were inoculated with the *F. graminearum* strain “H-3”, which produces DON, by pipetting 10 μL of a fungal suspension (1 × 10^5^ macroconidia·mL^−1^) between the lemma and palea of each floret. At least 20 florets per spike were inoculated on the same day. At the same time, control plants were mock inoculated with 10 μL of distilled water. The inoculated spikes were covered with a plastic bag for 72 hours, and any contact between the bag and the spike was avoided. Temperature and moisture content in the green house were maintained at 25 °C and 50%, respectively. For RNA extraction, inoculated spikelets of six spikes per genotype/treatment/time point were sampled, and the time points were 0, 1, 3, 5 and 7 days after inoculation (dai). Samples were immediately frozen in liquid nitrogen and stored at −80 °C. For the microarray analysis, samples from three biological replicates were taken at 3 and 7 dai.

### 4.3. Mycotoxin Analysis 

DON and D3G were estimated by liquid chromatography coupled to tandem mass spectrometry (LC-MS/MS) as explained by Nakagawa *et al*. [31] with slight modification. This method was well-standardized and validated with many laboratories. In brief, the *F.g* inoculated wheat florets were taken, ground, and its aliquot (0.1 g) was fortified with the internal standard (0.2 mg/L verrucarol in acetonitrile) so that its concentration was adjusted as 0.1mg/kg. After being kept in the refrigerator for more than 12 h, the florets powder was extracted with 8 ml of acetonitrile/water (80/20, *v*/*v*), and 0.08 mL of acetic acid (>99.9%) by blending a homogenizer for 5 min. The resulting slurry was centrifuged at 2,000× *g* for 10 min, and a portion of the supernatant (6 mL) was loaded directly on a multifunctional column InertSep VRA-3 (GL Sciences, Tokyo, Japan, Part No.5010-68142) for purification. After discarding the first 3 mL of solvent coming off the column, a 1.6 mL aliquot of the remainder was dried under a nitrogen gas stream at 40 °C. The residue was re-dissolved in 0.1 mL of acetonitrile/water/acetic acid (5/94/1, *v*/*v*/*v*) for LC-MS/MS analysis. The LC-MS/MS analysis was conducted as previously reported except that D3G by monitoring [D3G+CH_3_COO]^−^ with precursor/product ions (*m/z*) of 517.1/456.9 (for quantification) and 517.1/58.9 (for qualification). The employment of internal standard (verrucarol) was apparently effective to ensure repeatability and reproducibility with sufficient recovery of each mycotoxin. The ratio of D3G and DON was estimated with its absolute value to see the trend of mycotoxin levels among three wheat genotypes. Student *t*-test was used for comparing the D3G/DON data among cultivars. As a cross-validation, the samples were analysed with/without internal standard and also using an iPhree column (Phenomenex, Torrance, CA, USA, Part No.8B-S133-TAK) to remove phospholipids.

### 4.4. RNA Extraction and cRNA Synthesis

Total RNA was extracted from collected samples, using a Nucleo Spin RNA plant kit (Macherey-Nagel, Düren, Germany), and then converted to cRNA and labelled using a Low Input Quick Amp Labelling Kit (Agilent Technologies, Santa Clara, CA, USA) and fluorophore Cyanine 3-cytidine triphosphate (cy3-CTP). The samples were cleaned using an RNeasy MinElute Cleanup Kit (Qiagen, Hilden, Germany). The evaluation of RNA quantity and quality was conducted using a NanoDrop ND-1000 spectrophotometer (Thermo Scientific, Wilmington, DE, USA) and 1.5% agarose gel electrophoresis.

### 4.5. Microarray Assay

A wheat 38k oligonucleotide DNA microarray (Agilent Technologies, Santa Clara, CA, USA) was used for the microarray analysis. The platform of the Gene Expression Omnibus (GEO) is GPL9805 in the National Center of Biotechnology Information (NCBI), and more detailed information on the 38k microarray is in Kawaura *et al.* [32]. The microarray assay was used to measure the changes in global gene expression among the three genotypes with and without (mock control) FHB infection at 3 and 7 dai. cRNA labelling with one color, hybridization and further washing was carried out according to the Agilent technical protocol. In total, 12 samples were hybridized and biologically replicated three times (12 samples × 3 replication = 36 samples). Gene intensities were extracted from the scanned images, and the data were analyzed using the Gene Spring 12.6 software (Agilent Technologies, Santa Clara, CA, USA). The microarray data were deposited in the NCBI Gene Expression Omnibus (GEO) database with Accession Number GSE59721.

### 4.6. Data Analyses

**Figure 7:**
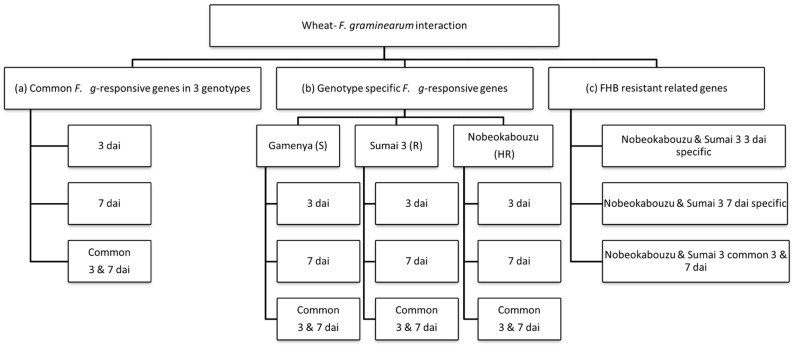
Flow chart of the data analysis. The expression of *F. graminearum*-responsive wheat genes was classified as: (**a**) common *F. graminearum*-responsive genes in wheat genotypes by comparative selection at 3 and 7 dai; (**b**) genotype-specific *F. graminearum*-responsive genes for susceptible, resistant and highly resistant wheat genotypes at 3 and 7 dai; and (**c**) FHB resistance-related genes at 3 and 7 dai. S, R and HR denote the susceptible, resistant and highly resistant cultivars, respectively.

After normalization and statistical analysis (Benjamin–Hochberg FDR, 2-way ANOVA with a *p* (treatment type and treatment time point) cut-off of 0.05 and a fold change ≥2), the data were grouped by Venn diagram to categorize the genes. The categories are (i) commonly-expressed genes to *F. graminearum* (*F. g*-responsive genes), which are differentially expressed genes against *F. graminearum* infection in three wheat genotypes; (ii) genotype-specific differentially expressed genes, which are responsive to *F. graminearum* in susceptible, resistant and highly resistant wheat genotypes at specific time points; and (iii) FHB resistance-related differentially expressed genes, which are identified by the selective comparison of resistant and highly FHB-resistant genotypes (Figure 7). The expressed genes were then assigned to 11 different gene classes derived from a combination of previous patho-transcriptomic studies [17,18,22,33]: (1) JA- and ET-related genes; (2) jasmonate-regulated proteins (JRP); (3) GDSL-lipases; (4) miscellaneous defense-related genes, such as the disease resistance-responsive protein family and the NBS-LRR disease resistance protein; (5) cysteine-rich antimicrobial peptides (AMPs), including serine-protease inhibitors; (6) proteolysis, including serine proteases; (7) peroxidases (POD); (8) genes related to cell wall defense, such as polygalacturonase-inhibiting proteins, xylanase inhibitors and glucan endo-1,3-β-glucosidase precursors; (9) genes involved in detoxification and secondary metabolism; (10) genes related to transcription and signaling; and (11) hormone (auxin, gibberellins, abscisic acid and salicylic acid) metabolism-related genes. To clearly visualize the molecular responses of the genotypes, the gene classes were categorized into three functional groups: (I) systemic defense-related genes, including genes that are known to play an important role in plant immunity by eliciting systemic defense (includes gene Classes 1, 2, 3, 4 and 11); (II) local defense-related genes, which are composed of genes that interact directly with the pathogen to avoid fungal spread (includes gene Classes 5, 6, 7 and 8); and (III) genes involved in detoxification, which includes those involved in detoxification processes and secondary metabolism (includes gene Classes 9 and 10). The functions of the probes and genes were predicted by BLAST searches (*E-*value, 1e^−10^), and further *in silico* mapping analysis was attempted with a Chinese Spring survey sequence of individual wheat chromosomes (http://wheat-urgi.versailles.inra.fr/Projects).

### 4.7. Quantitative Real-Time PCR (qPCR) Assay

The differential gene expression among genotypes was confirmed using a qPCR assay. cDNA was synthesized from RNA samples from 0, 1, 3, 5 and 7 dai using an RNA PCR kit (AMV) ver. 3.0 (TAKARA, Japan). Six genes were selected, and their details are shown in Table 1. The primers for the qPCR were designed using QuantPrimer software [34] (http://www.quantprime.de) and were based on published gene sequences (National Center of Biotechnology Information-NCBI). A Brilliant III Ultra-Fast SYBR Green QPCR Master Mix kit (Agilent Technologies, Santa Clara, CA, USA) was used, and the PCR reaction was carried out with an Mx Pro3000 Real-time PCR (Stratagene, Agilent Technologies Division, Santa Clara, CA, USA). Each reaction contained 10 μL of 2× SYBR Green Master Mix, 2 μL of 100 ng/μL cDNA and 1 μL of 20 ρM forward and reverse primer in a final volume of 20 μL. The following thermal profile was used: 2 min at 50 °C, 10 min at 94 °C, 40 cycles of 45 s at 94 °C, 45 s at an annealing temperature of 65 °C and 45 s at 72 °C. All cDNA samples from the time points for each treatment were simultaneously amplified in one PCR plate and replicated twice. The expression of the target gene was quantified using the comparative 2^−ΔΔCt^ method. The expression of each target gene is presented as the fold change normalized to the reference gene, GAPDH, and relative to the untreated mock-infected control sample.

**Table 1:** Details of the primers used for the qPCR analysis.

Probe ID	Gene description	Accession number	Sequence (5'→3')
15510	UDP-glycosyltransferase	AK367614.1	F-GAGGAGGTTGCTAAGAAGGTCAGG
			R-ACCAGCCTTGCCAATGTCAC
8976	Multidrug resistance associate protein	AF532601.1	F-TCGAATCACCTCGGTCCTTCATAG
			R-ACAGCCATGCCATTGTCAAGGAG
23363	Plant thionin family protein	AK330802.1	F-CGAGGACCAAATCCATCAACCAAG
			R-AACACACTATCACACCCTTGAAGC
8436	Gamma-thionin family protein	BT009066.1	F-TTGCGGCTTCAATTAGTTTGCG
			R-TCAGCACAAGACAACAAAGAGGAG
19329	Peroxidase	X56011.1	F-CCTGCCAGGCTTTACATCTAGC
			R-TCGTAAGGAGGCCCTTGTTTCTG
7118	Monoglyceride lipase	AK332942.1	F-TCGATCACCCGATTGTCGTCAC
			R-AACTGGAACGTCATGCTTACCG
	Β-Actin		F-CCTTCCACATGCCATCCTTC
			F-GCTTCTCCTTGATGTCCCTTAC

Note: Wheat house-keeping gene *GADPH* is the control used for normalization. The number under Probe ID is as listed in the custom wheat array.

## 5. Conclusions

Based on our results, we revealed the similarities and differences in the molecular responses of three genotypes against *F. graminearum* at 3 and 7 dai. In the case of Nobeokabouzu, the resistance is due to the expression of abundant gene classes belonging to detoxification (Group III) and systemic defense (Group I), in the early stage of infection. After the first fungal attack, the plant starts to express defense-related genes that are induced by hormone signaling and activates systemic immunity to avoid fungal development inside the host. Alternatively, the genes involved in detoxification start to pump mycotoxins from the cell and, thus, inhibit fungal development. The early expression of these genes can interrupt fungal development and infection in Nobeokabouzu. In the case of Sumai 3, the resistance is mainly due to the expression of local defense-related genes in the late stage of infection. The results also allowed us to visualize the relationship between the molecular response and the phenotypical FHB symptoms at different time points. In the case of Gamenya, the minimum constitutive expression of defense-related genes allowed for quick infection and necrosis of the inoculated florets. In the case of Sumai 3 at 3 dai, however, we observed brownish necrosis in the inoculated florets, because of the lower expression of the local defense-related genes compared to Nobeokabouzu. However, high expression of those genes at 7 dai stopped the progression of the disease by blocking its spread along the spike. In highly FHB-resistant Nobeokabouzu, a few inoculated florets showed limited, brownish necrosis, but the plants were almost symptomless at both treatment time points. This clearly confirmed the early expression of local, systemic- and detoxification-related genes that avoided initial infection, as well as disease spread.

## Supplementary Materials

Supplementary materials can be accessed at: http://www.mdpi.com/2072-6651/7/2/0604/s1.
